# A theoretical interpretation of diffusion weighted and intravoxel incoherent motion imaging for cerebrospinal fluid flow

**DOI:** 10.1002/mrm.70062

**Published:** 2025-09-04

**Authors:** Tomohiro Otani, Yoshitaka Bito, Shigeki Yamada, Yoshiyuki Watanabe, Shigeo Wada

**Affiliations:** ^1^ Department of Mechanical Science and Bioengineering The University of Osaka Graduate School of Engineering Science Osaka Japan; ^2^ Department of Diagnostic Imaging Hokkaido University Graduate School of Medicine Hokkaido Japan; ^3^ Department of Neurosurgery Nagoya City University Graduate School of Medical Science Aichi Japan; ^4^ Interfaculty Initiative in Information Studies/Institute of Industrial Science The University of Tokyo Tokyo Japan; ^5^ Department of Radiology Shiga University of Medical Science Shiga Japan

**Keywords:** cerebrospinal fluid, diffusion, intravoxel incoherent motion, intravoxel standard deviation, neurofluids

## Abstract

**Purpose:**

Diffusion‐weighted imaging (DWI) and intravoxel incoherent motion (IVIM) imaging are well‐established approaches for evaluating cerebrospinal fluid (CSF) flow in subarachnoid and perivascular spaces, and have recently been applied to study ventricular CSF flow. However, DWI does not directly measure flow velocity, and the physical implications of DWI measurements are unclear. This study aimed to provide a theoretical interpretation of the DWI and IVIM imaging of CSF flow velocity fields.

**Theory:**

The general semi‐analytical form of the signal attenuations caused by fluid flow and the resultant apparent diffusion coefficient were derived from the Bloch–Torrey equation for arbitrary b values.

**Methods:**

The fundamental properties of the signal attenuation in laminar flow velocity fields were investigated. A Monte Carlo simulation of the IVIM parameter estimation was performed based on these signal attenuations, taking background noise into consideration.

**Results:**

The developed theoretical framework indicates that signal attenuations in DWI detect intravoxel flow velocity standard deviations ranging from approximately 0.1 to 10 mm/s within the range of practical scan parameter settings. The lower bounds of the DWI flow profiles appeared where the flow effect was an order of magnitude lower than the molecular diffusion effects, even when b increased. The IVIM fitting parameters reflected the flow effects of the signal attenuations despite an inconsistency with the original IVIM model assumptions.

**Conclusion:**

The physical implications of signal attenuation in DWI have been theoretically clarified. This framework provides a useful basis for understanding CSF flow dynamics and considering appropriate imaging settings.

## 1 INTRODUCTION

Intracranial cerebrospinal fluid (CSF) plays essential roles in the transport of molecular signals and the clearance of waste products^1^, and its flow characteristics have gained much attention. Magnetic resonance imaging (MRI) provides a unique tool for the non‐invasive evaluation of subject‐specific CSF characteristics, and various approaches to CSF flow imaging have been established,^2^ such as phase‐contrast (PC) imaging,^3^, ^4^ diffusion‐weighted imaging (DWI),^5^, ^6^, ^7^ spin‐labeling^8^, ^9^, ^10^ and functional MRI^11^.

Because of the slow flow properties of intracranial CSF flow, its imaging presents severe technical difficulties. In this context, DWI and the apparent diffusion coefficient (ADC) provide a promising approach.^6^, ^12^, ^13^ In particular, low‐b DWI is thought to be able to capture the signal attenuation originating from CSF flow while avoiding molecular diffusion effects.^14^, ^15^, ^16^, ^17^ Furthermore, several studies have applied DWI model‐based analysis using the intravoxel incoherent motion (IVIM) model^18^ to CSF flow imaging in the brain parenchyma and subarachnoid spaces.^19^, ^20^, ^21^, ^22^, ^23^ This method assumes two compartments within a voxel (perfusion and diffusion) and estimates the perfusion fraction.^18^, ^24^, ^25^ In recent studies, the IVIM model and its parameters have been extended to ventricular CSF flow analyses.^26^, ^27^, ^28^, ^29^

A key limitation of DWI in the context of fluid flow imaging is that it does not directly measure flow velocity,^6^ and both ADC and IVIM models rely on assumptions that may not hold in single‐component flows. Nevertheless, Jang et al.^30^ reported strong correlations between ADC and peak flow velocity according to computational simulations, and thus DWI may reflect certain properties of intravoxel fluid flow. Bito et al.^14^ investigated the theoretical properties of the ADC and showed that the limit of the ADC with sufficiently low b is a function of the variance of intravoxel flow velocity. Equivalent formulations were developed in both DWI modeling^31^ and PC imaging,^32^ and have been widely used for estimating turbulent kinetic energy,^33^ particularly in PC imaging, where the intravoxel flow distribution is typically assumed to follow a normal distribution. Combining concepts from both DWI‐ and PC‐based formulations could provide a general theoretical form for the ADC for arbitrary b, and could establish a theoretical framework for interpreting the DWI and IVIM imaging of CSF flow fields.

The study aimed to theoretically interpret DWI and IVIM signals for slow flows, such as intracranial CSF flow. A general semi‐analytical form of the signal attenuations by the diffusion gradient pulses characterized by b was derived from the Bloch–Torrey equation. The fundamental properties of the signal attenuations and detectable ranges of intravoxel flow velocity distributions were estimated considering clinically practical conditions, and the physical meaning of the IVIM model fitting for single CSF flow was demonstrated.

## 2 THEORY

### 2.1 Signal attenuation of magnetization

A three‐dimensional domain is defined in a Cartesian coordinate system (o−xyz) with the static magnetic field directed along the z‐axis. Considering a magnetization vector $m={\left(m_{x},m_{y},m_{z}\right)}^{⊤}$ in the target fluids filling in this domain, the Bloch–Torrey equation for the transverse component of m ($m=m_{x}+im_{y}$) in a rotating frame is given by,

(1) $$\frac{\partial m}{\partial t}=-i\gamma (G\cdot x)m-\frac{m}{T_{2}}+D\nabla^{2}m,$$

where γ is the gyromagnetic ratio, G(t) is the spatial gradient magnetic field along the z‐axis, x(t) is the position vector of m, $T_{2}$ is the transverse relaxation time, and D is the diffusion coefficient of the target fluids. The position vector x(t) is defined as

(2) $$x(t)=x(0)+\int_{0}^{t}v(x;\tau )d\tau$$

where v(x;t) is the flow velocity vector expressed as a smooth and continuous spatiotemporal function. The semi‐analytical solution of m can be derived as^34^, ^35^

(3) $$\begin{array}{ll} m(x;t) & =C\;\underset{\text{relaxation}}{\underbrace{\exp\left(-\frac{t}{T_{2}}\right)}}\\ & \;\cdot \underset{\text{diffusion}}{\underbrace{\exp\left[-\gamma^{2}D\int_{0}^{t}{\left(\int_{0}^{t^{\prime }}G(\tau )d\tau \right)}^{2}dt^{\prime }\right]}}\\ & \;\cdot \underset{\text{precession}}{\underbrace{\exp\left(-i\gamma \int_{0}^{t}x(\tau )\cdot G(\tau )d\tau \right)}}, \end{array}$$

where C is a constant.

From here, G(t) and v(x;t) along the same direction are considered, and these values are denoted as scalar values G(t) and v(x;t), respectively. Here, the scalar v is defined as the projection of the flow velocity vector field along the direction of the diffusion gradient. Furthermore, v(x;t) is assumed to be constant during the diffusion gradient pulse T. Using these assumptions, the signal attenuation is expressed as the change ratio of m with and without the gradient pulse, given by^34^

(4) $$\frac{m}{m_{0}}=\exp(-bD)\cdot \exp(-ikv),$$

where

(5) $$b=\gamma^{2}\int_{0}^{T}{\left(\int_{0}^{t}G(\tau )d\tau \right)}^{2}dt,\;k=\gamma \int_{0}^{T}tGdt,$$

and $m_{0}$ is the transverse magnetization without the gradient pulse (G(t)=0). If the Stejskal–Tanner pulse is applied, these values are uniquely determined as $b=\gamma^{2}G^{2}\delta^{2}\tau_{d}$ and k=γGδΔ, where δ and Δ are the duration and separation of the gradient pulse, respectively, and $\tau_{d}=\Delta -\frac{\delta }{3}$. Here, the relationship between k and b is given by

(6) $$k^{2}=\frac{b\Delta^{2}}{\tau_{d}}.$$

From here, the Stejskal–Tanner pulse is assumed as the gradient pulse unless otherwise noted.

### 2.2 Intravoxel signal attenuation

To extend Equation 4 to the total signal attenuation in a voxel, an integral form with respect to flow velocity was derived in PC^32^ and DW^14^, ^31^ imaging studies, as follows.

(7) $$\frac{S}{S_{0}}=\exp(-bD)\int_{-\infty }^{\infty }f(v)\exp(-ikv)dv,$$

where signals S and $S_{0}$ are summations of m and $m_{0}$ distributed in a voxel, respectively, and f(v) is the probability density function of v. Since the above integral is equivalent to the Fourier transform of f(v) ^32^ (i.e., the characteristic function^36^), Equation 7 can be rewritten as

(8) $$\frac{S}{S_{0}}=\underset{\text{signal attenuation}}{\underbrace{\exp(-bD)\hat{F}(k)}}\cdot \underset{\text{phase shift}}{\underbrace{\exp(-ikv_{m})}},$$

where $\hat{F}(k)$ is the characteristic function of $\hat{f}(v)=f(v-v_{m})$ by the shift theorem, and $v_{m}$ is the mean of f(v) extracted in PC imaging. From Equation 8, the ADC is expressed as

(9) $$\begin{array}{ll} \text{ADC} & :=-\frac{1}{b}\ln\left|\frac{S}{S_{0}}\right|=D-\frac{1}{b}\ln|\hat{F}(k)|,\\ & ∵\;\left|\frac{S}{S_{0}}\right|=\exp(-bD)\hat{F}(k). \end{array}$$

Thus, the ADC can be understood as a summation of the diffusion coefficient D and the intravoxel flow distributions independent of $v_{m}$. Since $|\hat{F}(k)|\leq 1$ ^36^ and b≥0, the ADC is positive.

#### Remark

Bito et al.^14^ pointed out that the limit of the ADC as b goes to zero is uniquely determined in arbitrary $\hat{f}(v)$, as follows.

(10) $$\underset{b\to 0}{\lim}\;\text{ADC}=D+\frac{\sigma_{v}^{2}\Delta^{2}}{2\tau_{d}},$$

where $\sigma_{v}^{2}$ is the variance of the intravoxel flow velocity. From Equation 8, this limit operation can be understood from the limit of $\hat{F}(k)$ as k goes to zero. Consider $\hat{F}(k)$ with small k,

(11) $$\begin{array}{ll} \hat{F}(k) & =\int_{-\infty }^{\infty }\hat{f}(v)\left(1-ikv-\frac{k^{2}v^{2}}{2}+O(k^{3})\right)dv\\ & \approx \int_{-\infty }^{\infty }\hat{f}(v)dv-ik\int_{-\infty }^{\infty }\hat{f}(v)vdv-\frac{k^{2}}{2}\int_{-\infty }^{\infty }\hat{f}(v)v^{2}dv\\ & =1-\frac{k^{2}\sigma_{v}^{2}}{2}, \end{array}$$

which is equivalent to that of a normal distribution with zero mean and variance of $\sigma_{v}^{2}$, such that

(12) $$\begin{array}{ll} \hat{F}(k) & =\exp\left(\frac{-k^{2}\sigma_{v}^{2}}{2}\right),\\ & =1-\frac{k^{2}\sigma_{v}^{2}}{2}+𝒪(k^{3}). \end{array}$$

Thus, substituting Equation 12 into Equation 9 leads to Equation 10 without a limit operator, such that

(13) $$\begin{array}{ll} \text{ADC} & =D-\frac{1}{b}\ln\left|\exp\left(\frac{-k^{2}\sigma_{v}^{2}}{2}\right)\right|\\ & =D+\frac{\sigma_{v}^{2}\Delta^{2}}{2\tau_{d}}. \end{array}$$

The correspondence of Equations 11 and 12 is commonly used in the proof of the central limit theorem (e.g., Reference [37]), where high‐order terms of the characteristic function are omitted as the number of trials increases.

### 2.3 $\hat{f}(v)$ and $\hat{F}(k)$ in laminar flow

The flow velocity field is assumed to be laminar and sufficiently slow such that it can be expressed as a low‐order polynomial function within a voxel. Furthermore, the flow field is assumed to be temporally constant, and the flow variance discussed in the following sections reflects only spatial variation.

Let us consider a local coordinate system defined within a voxel (right‐handed), where the origin is placed at the voxel center and the orthogonal unit vectors are aligned with the voxel edges. Assuming that v varies linearly in space, it is given by

(14) $$\begin{array}{ll} v(x,y,z) & =v_{0}+\frac{\partial v}{\partial x}x+\frac{\partial v}{\partial y}y+\frac{\partial v}{\partial z}z\\ & =v_{0}+a_{1}x+a_{2}y+a_{3}z, \end{array}$$

where $v_{0}$ is the velocity at the voxel center, which coincides with the spatial average of v within the voxel, and $a_{i}$ (i=1,2,3) is the spatial velocity gradients along the respective coordinate directions. Here, the voxel size is given by $\left[-L_{1},L_{1}]\times [-L_{2},L_{2}]\times [-L_{3},L_{3}\right]$ and the range of v is normalized to $\left[-v_{d},v_{d}\right]$.

**FIGURE 1:**
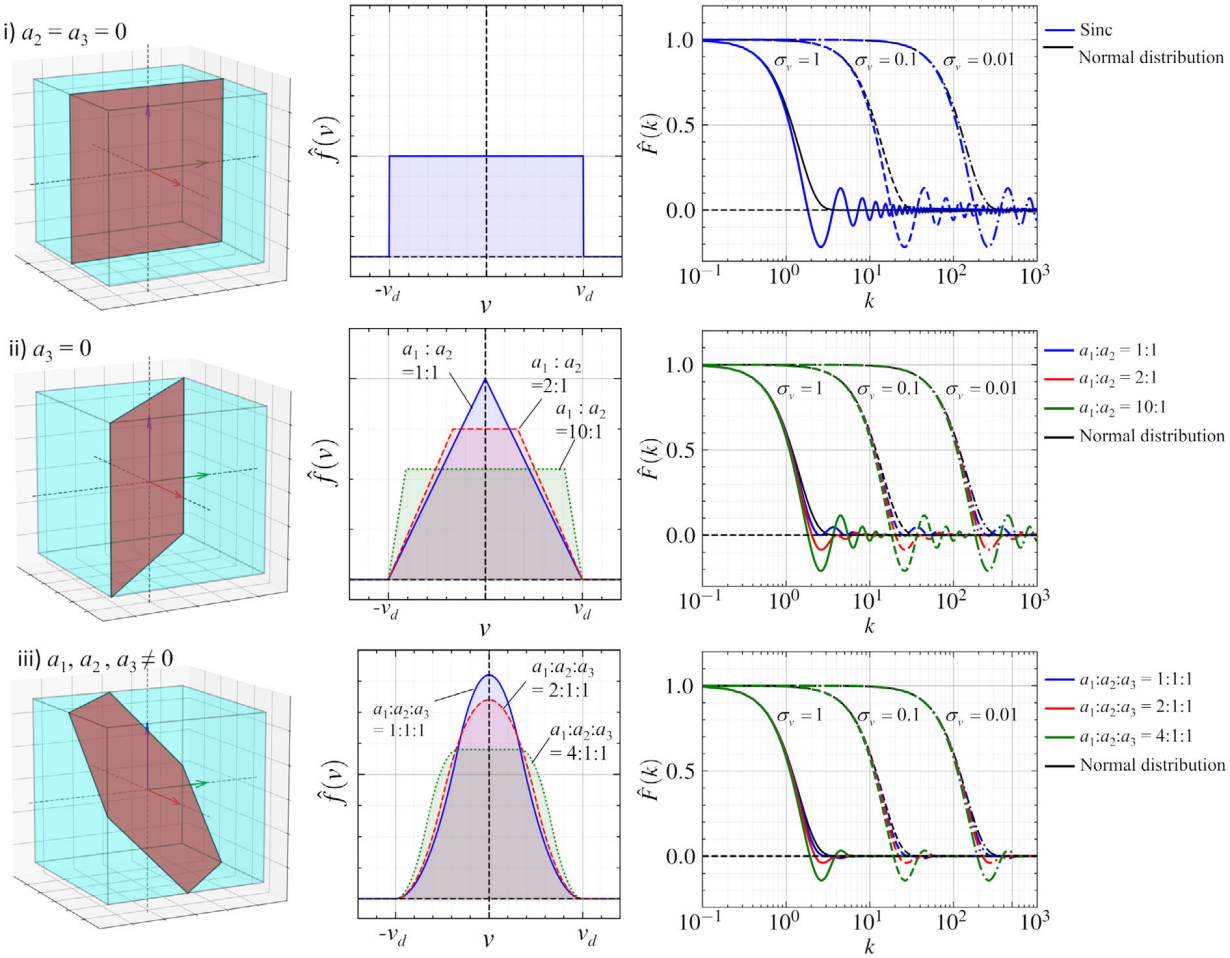
Representative cases of v iso‐surfaces in an isotropic voxel (left), corresponding probability density functions $\hat{f}(v)$ of the flow velocity $v\in [-v_{d},v_{d}]$ (center), and the characteristic function $\hat{F}(k)$ with $\sigma_{v}=1,0.1$, and 0.01 (right) in cases that (i) the velocity gradient is negligible in two‐directions, (ii) the velocity gradient is negligible in one direction, and (iii) the velocity gradient is comparable in all directions.

Following the above definition, $\hat{f}(v)$ is defined based on a cross‐sectional area of the v iso‐surface in the voxel (Figure 1 (left)). First, if two components are negligible (e.g., $a_{2}=a_{3}=0$), the $\hat{f}(v)$ is a uniform distribution denoted as ${\hat{f}}_{\text{1D}}(a_{1},L_{1})$, given by

(15) $$\begin{array}{ll} {\hat{f}}_{\text{1D}}(a_{1},L_{1}) & =\frac{1}{2a_{1}L_{1}},\\ v_{d} & =a_{1}L_{1}. \end{array}$$

Thus, the corresponding $\hat{F}(k)$ is a sinc function, such that

(16) $$\hat{F}(k)=\text{sinc}(ka_{1}L_{1}).$$

In general cases ($a_{1},a_{2},a_{3}\neq 0$), $\hat{f}(v)$ is expressed as a convolution integral^37^ of the uniform distribution ${\hat{f}}_{\text{1D}}$ assigned to each axis of interest, such that

(17) $$\begin{array}{ll} \hat{f}(v) & ={\hat{f}}_{\text{1D}}(a_{1},L_{1})*{\hat{f}}_{\text{1D}}(a_{2},L_{2})*{\hat{f}}_{\text{1D}}(a_{3},L_{3}),\\ v_{d} & =\overset{3}{\underset{i=1}{\sum }}\;a_{i}L_{i}, \end{array}$$

where ∗ denotes the convolution integral. The variance of flow velocity $\sigma_{v}^{2}$ is also generalized as

(18) $$\sigma_{v}^{2}=\frac{{\left(a_{1}L_{1}\right)}^{2}+{\left(a_{2}L_{2}\right)}^{2}+{\left(a_{3}L_{3}\right)}^{2}}{3}.$$

Finally, the corresponding $\hat{F}(k)$ is given by

(19) $$\hat{F}(k)=\overset{3}{\underset{i=1}{\prod }}\text{sinc}(ka_{i}L_{i}).$$

Figure 1 (center and right) shows representative examples of $\hat{f}(v)$ curves with constant $v_{d}$ and $\hat{F}(k)$ curves with $\sigma_{v}=0.01,0.1$, and 1. When $\hat{f}(v)$ follows a uniform distribution, $\hat{F}(k)$ becomes a sinc function and exhibits oscillatory behavior around zero as k increases. As velocity gradients become more multidirectional and comparable in magnitude, $\hat{f}(v)$ approaches a triangular‐like distribution, and the oscillations in $\hat{F}(k)$ become weak. Curves of $\hat{F}(k)$ between original and corresponding normal distributions with the same $\sigma_{v}$ are closed when $\hat{F}(k)$ is higher than 0.5, regardless of flow distributions. Although $\hat{F}(k)$ in normal distributions mildly decay compared to those of the original $\hat{F}(k)$, these differences are at most 4% in the case of $a_{1}=a_{2}=a_{3}$. This excellent agreement is reasonable because the multiple convolution integrals of the probability density function, which is independent and identically distributed, approach the normal distribution based on the central limit theorem.^37^

## 3 METHODS

The fundamental properties of the intravoxel signal attenuation and the physical interpretation of the DWI and IVIM imaging of the laminar flow were investigated with consideration of the CSF flow imaging. We set D of the CSF to $3.0\times 10^{-3}$ mm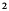/s as the diffusion coefficient of pure water at 37°C.^38^ For modeling $\hat{F}(k)$ in Equation 19, we considered a simplified case with isotropic voxel ($L_{1}=L_{2}=L_{3}$) and velocity gradients ($a_{1}=a_{2}=a_{3}$), which represents a fully three‐dimensional flow distribution in a representative manner.

The DWI signal attenuations were evaluated under ideal and noise‐affected conditions. In the latter case, the background noise of $\left|S/S_{0}\right|$ was modeled using a Rician distribution,^39^ with the signal‐to‐noise ratio (SNR) set to 20 as a representative value. Assuming that the true MRI signal is zero, the mean value of the background noise $\bar{\epsilon }$ is given by^39^

(20) $$\bar{\epsilon }=\sigma \sqrt{\pi /2}$$

where σ=1/SNR, assuming that the $S_{0}$ is normalized to 1. This value $\bar{\epsilon }$ was used to evaluate the non‐zero baseline in the low‐signal regime (i.e., noise floor^40^).

First, the ranges of $\sigma_{v}$ detectable using DWI were investigated. To consider the relative extents of the intravoxel flow velocity distributions with respect to the diffusion effect, we introduced the scaling factor α≥0 from Equation 10 and rewrote the ADC (Equation 13) in the sufficiently low‐b case (b→0), as follows.

(21) $$\begin{array}{ll} & \frac{\sigma_{v}^{2}\Delta^{2}}{2\tau_{d}}=\alpha D, \end{array}$$

(22) $$\begin{array}{ll} & \underset{b\to 0}{\lim}\;\text{ADC}=(1+\alpha )D. \end{array}$$

For the evaluation, we set α=0 (pure diffusion), 0.1,1,10,100, and 1000. Furthermore, the sensitivities of δ and Δ were evaluated in the case of $\sigma_{v}=0.4$ mm/s and b=100 s/mm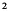. Both δ and Δ were set from 0 ms to 60 ms, and Δ≥δ were set according to the definitions.

Next, the IVIM model fitting was applied to the signal attenuations of the representative three cases of α = 0.1, 1, and 10. The IVIM model is defined as the following bi‐exponential function^18^:

(23) $${\left|\frac{S}{S_{0}}\right|}_{\text{IVIM}}:=\underset{\text{perfusion}}{\underbrace{f_{\text{VOF}}\exp(-bD_{p})}}+\underset{\text{diffusion}}{\underbrace{\left(1-f_{\text{VOF}})\exp(-bD_{d}\right)}},$$

where $D_{p}$ is the pseudo diffusion coefficient originating from the perfusion effect, $D_{d}$ is the molecular diffusion coefficient estimated in this model, and $f_{\text{VOF}}$ is the volume fraction of the perfusion components ($f_{\text{VOF}}\in [0,1]$). To consider practical conditions, we extracted signal attenuations at b=0,50,100,250,500, and 1000 s/mm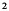 based on Reference [28]. Based on the Rician noise characteristics described above, Monte Carlo simulations of the IVIM model fitting were performed with a number of trials of $10^{4}$ in each case of α. These fits were computed using the curve‐fit algorithm implemented in SciPy^41^ with the constraints of $D_{p}>D_{d}\geq 5\times 10^{-5}$ mm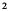/s for stability.

## 4 RESULTS

**FIGURE 2:**
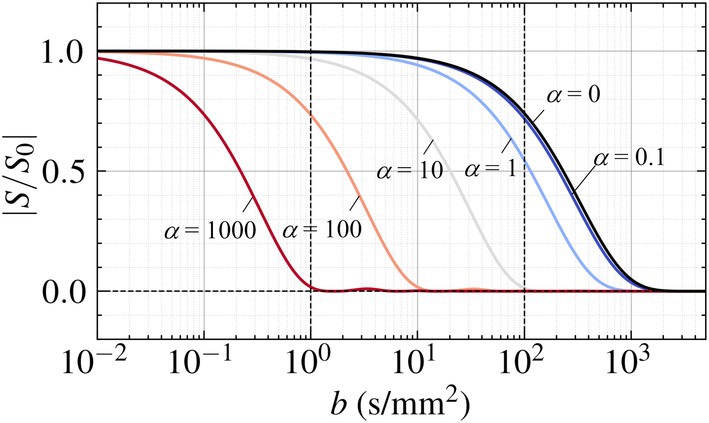
Degree of signal attenuation with increasing b in representative cases with intravoxel velocity disturbances of α = 0 (diffusion only), 0.1, 1, 10, 100, and 1000.

Figure 2 shows signal attenuation curves with increasing b in representative cases of α. The signal of α = 0.1 was almost equal to that of α=0 (pure diffusion) at b<10 s/mm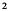, was slightly lower at b>10 s/mm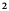, and then approached zero at b of around 1000 s/mm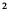. These signal curves shifted to lower b with increasing α, and that of α = 1000 decayed at low b from 0.01 to 1 s/mm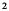 and approached zero at b≈1 s/mm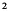.

**FIGURE 3:**
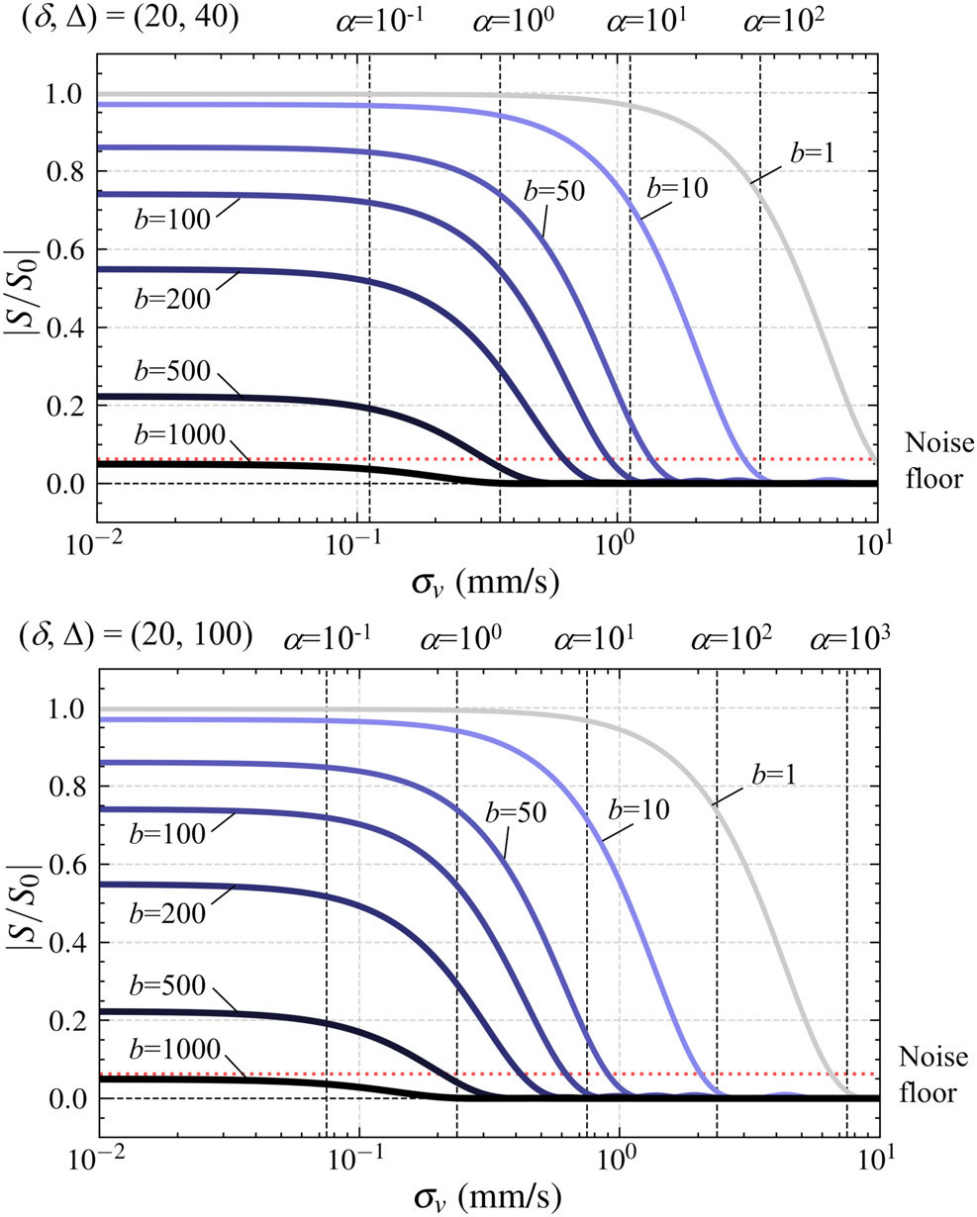
Sensitivities of $\sigma_{v}$ on signal attenuation in the ranges of b = 1, 10, 50, 100, 200, 500, and 1000 at (δ, Δ) = (20, 40) ms (20, 100) ms, as representatives. Dashed lines in red in horizontal directions show the mean of background noises (noise floor) estimated as a Rician distribution (SNR = 20).

For practical interpretation of the detectable velocity range, the signal attenuation curves with respect to $\sigma_{v}$ are summarized for different b at (δ,Δ) = (20, 40) ms and (20, 100) ms, as shown in Figure 3. In the case of (δ,Δ) = (20, 40) ms, the signal decreased in $\sigma_{v}$ of 𝒪(1) mm/s at b=1 s/mm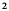. The curves were shifted to lower $\sigma_{v}$ with increasing b, while the signal baselines (e.g., those at $\sigma_{v}=0$ mm/s) also became lower and reached values comparable to the noise floor ($\bar{\epsilon }\approx 0.06$) at b=1000 s/mm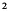. In addition, the signal attenuation curves were shifted to relatively low $\sigma_{v}$ as Δ increased, as shown in the case of (δ,Δ) = (20,100) ms.

**FIGURE 4:**
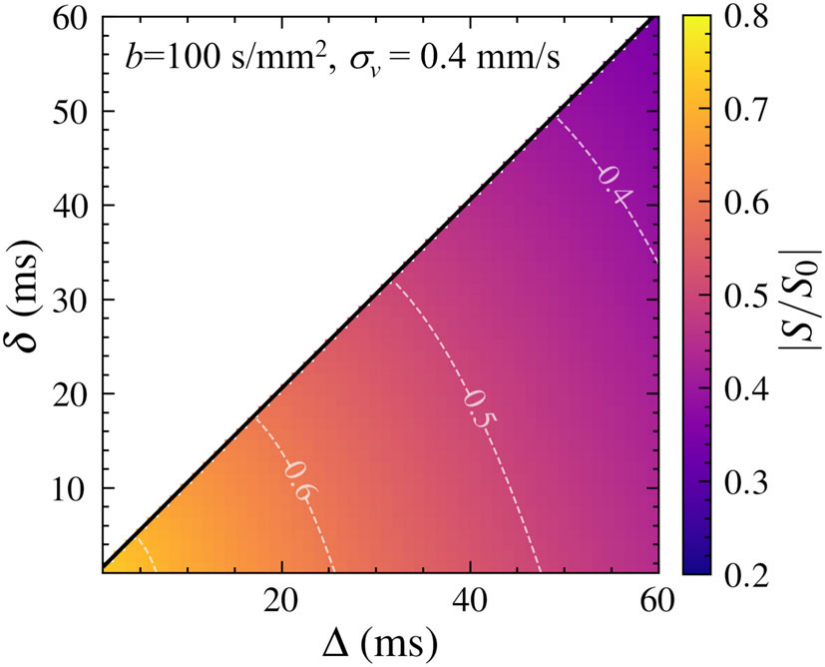
Sensitivities of δ and Δ on signal attenuations in the ranges of δ∈[0,60] and Δ∈[0,60] at b = 100 s/mm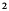 and $\sigma_{v}$ = 0.4 mm/s.

The effects of δ and Δ on the extent of signal attenuation are summarized in Figure 4 in the representative case of $\sigma_{v}$ = 0.4 mm/s and b = 100 s/mm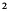. The signal decreased monotonically, while the degree of attenuation became mild with increasing δ and Δ, and ranged from approximately 0.7 to 0.3.

**FIGURE 5:**
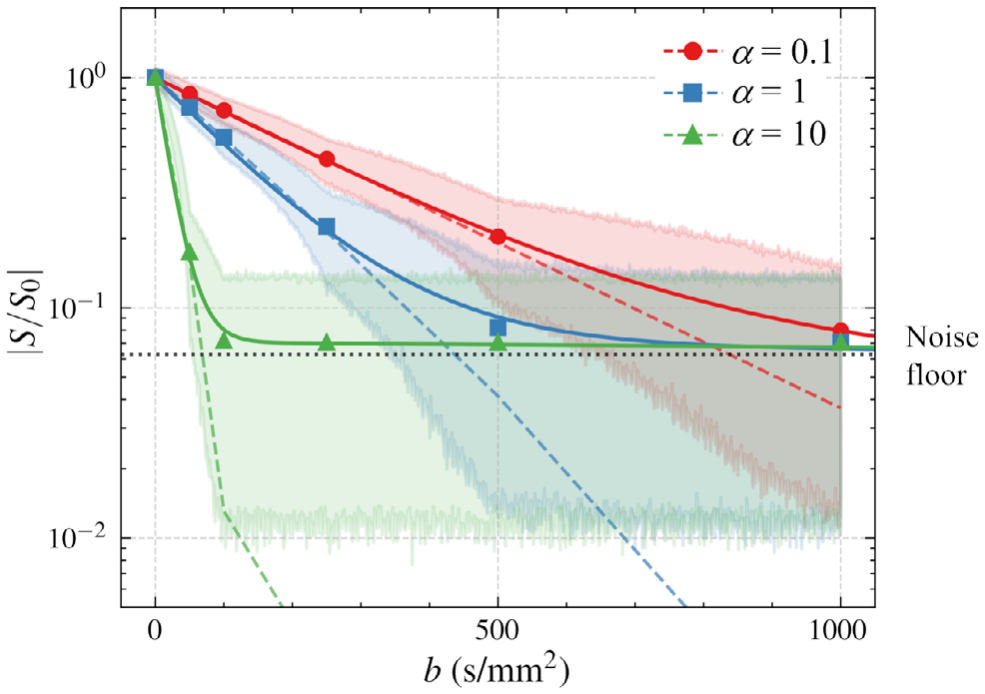
Signal attenuations at b = 0, 50, 100, 250, 500, and 1000 in cases of α = 0.1, 1, and 10. Shaded domains show the range of noise of SNR = 20, and the dotted line shows the mean of the noise (noise floor). Solid and dashed lines show the fitting curves of the IVIM model (Equation 23) on the mean signal values and original signal attenuation curves without noise, respectively. The kink observed in the dashed line for α=10 near $|S/S_{0}|=10^{-2}$ is attributed to the oscillatory behavior of the $\hat{F}(k)$ in the signal attenuation curve.

Finally, the IVIM model was fitted to the signal attenuation curves obtained at multiple b‐values. Figure 5 shows IVIM curves fitted to the mean of the signals with Rician noise (solid lines) and the original noise‐free signal attenuations (dashed lines) in three representative cases of α= 0.1, 1, and 10. As the b increased, the signals approached the noise floor, and thus resulted in bi‐exponential‐like curves whose characteristics depend on the flow effects α. In this setting, the IVIM parameters (particularly $f_{\text{VOF}}$ and $D_{p}$) reflected the slope of flow‐induced signal attenuation in the low‐b range. The corresponding values and their variability are provided in Supplementary 1.

## 5 DISCUSSION

This study aimed to extend the existing theory of the DWI of fluid flow with sufficiently low b ^14^ to the generalized theoretical framework in arbitrary b by combining knowledge on both DWI^14^ and PC^32^ imaging. The semi‐analytical ADC expression (Equation 9) consists of the molecular diffusion term and a flow term, determined solely by the velocity distribution and independent of mean velocity. From this theory, the ADC limit with sufficiently low b ^14^ can be understood from the property of the characteristic function $\hat{F}(k)$.

Using the theoretically derived signal attenuation, we investigated the range of CSF flow velocities detectable under clinically relevant scan settings. The framework demonstrated that DWI is capable of detecting signal attenuation originating from flow effects when the velocity standard deviation $\sigma_{v}$ falls within approximately 0.1–10 mm/s (Figure 3). If the molecular diffusion coefficient D is known a priori (e.g., the imaging target is pure fluids such as CSF), the flow contribution can be extracted by subtracting the molecular diffusion effects from the signal attenuations. Moreover, the signal intensity is influenced not only by b, but also by δ and Δ ^15^ (Figure 4). Thus, to estimate flow‐related contributions more robustly, one possible strategy is to vary δ and Δ while keeping b constant. This allows the diffusion‐related component of the signal to remain fixed, while the flow‐related component varies according to changes in the gradient waveform. Therefore, signal differences across multiple (δ,Δ) combinations at constant b could help extract intravoxel flow variance. Although further validation with actual MRI sequences and implementation would be required, this theoretical framework provides a promising basis for future development of DWI‐based flow quantification.

Furthermore, we explored how IVIM model fitting behaves when applied to single‐component flow fields, despite the original IVIM model being designed for two‐compartment systems. Under noise‐affected conditions, the signal attenuation curves approached a bi‐exponential‐like shape due to the noise floor (Figure 5), and the fitted IVIM parameters (particularly $f_{\text{VOF}}$ and $D_{p}$) reflected the slope of the flow‐induced signal attenuation in the low‐b region (Supplementary 1). Although the fitted parameters are not consistent with their original physical meaning, these findings may help in understanding recent IVIM studies on ventricular CSF flow,^26^, ^27^, ^28^, ^29^ where a single dominant flow component is likely present.

This study has three main limitations and perspectives for future work. The first is the assumption of isotropic velocity gradients ($a_{1}=a_{2}=a_{3}$), which was adopted to simplify the modeling and enable tractable analysis of signal attenuation behavior. While the signal attenuation curves are consistent with same $\sigma_{v}$ when $\hat{F}(k)>0.5$ regardless of the flow distribution (Figure 1), anisotropic flow profiles can induce oscillatory features in $\hat{F}(k)$, particularly in low‐signal regimes (Figure 1). As these regimes are often dominated by low SNR (Figure 2), further investigation would be needed to fully capture the impact of anisotropy under realistic acquisition conditions. The second is that we assumed the flow velocity to be sufficiently slow such that motion artifacts due to magnetization advection^42^ could be neglected. Since the repetition time of general DWI using echo‐planar imaging is on the order of 𝒪(1) seconds, this assumption may be critical for relatively high‐velocity flow fields.^43^ In such cases, the transport of magnetization over the duration of the pulse sequence may introduce significant artifacts, and theoretical modeling of these conditions becomes more challenging. The third is the assumption of steady flow. For ventricular CSF flow, the velocity is typically slow and synchronized with the cardiac cycle, and thus, the unsteady effects can be reduced by ECG‐gated DWI acquisition. However, in more general cases involving arbitrary time‐varying flows, unsteady effects may lead to non‐negligible artifacts. These effects arise from the steady‐flow assumption used in the derivation of b (Equation 4) and are similar to temporal misregistration artifacts observed in phase‐contrast MRI under similar assumptions.^44^ To address the above limitations, computational simulation of flow MRI under unsteady and anisotropic flow conditions^45^, ^46^ would be useful in future work. By modeling the spatiotemporal evolution of magnetization in arbitrary flow velocity fields, such simulations may help interpret the resulting DWI signals and the associated artifacts.

## 6 CONCLUSIONS

This study developed a general theoretical framework to understand the physical implications of DWI and IVIM imaging with arbitrary b. According to this theory, DWI can detect intravoxel flow velocity standard deviations ranging from 0.1 to 10 mm/s under practical conditions. Furthermore, the IVIM parameter fits of the single flow domain provide the effects of the intravoxel flow velocity distribution, although the original meaning of the IVIM model is inconsistent in this situation. These examples successfully highlight the usefulness of the developed theoretical framework, and therefore, we expect that this framework can provide attractive insights for understanding the DWI of fluid flow, help parameter tuning to detect the preferred flow velocity range, and assist in the development of further advanced imaging protocols.

## CONFLICT OF INTEREST STATEMENT

The authors declare no potential conflict of interest.

## FUNDING INFORMATION

This work was partially supported by Japan Society for the Promotion of Science Grants‐in‐Aid for Scientific Research (Grant Nos. 23K11830, 24K02408, 24K02557, 25K15857, and 25K03452); Program for Promoting Research on the Supercomputer Fugaku (Development of human digital twins for cerebral circulation using Fugaku) funded by the Ministry of Education, Culture, Sports, Science and Technology (Grant No. JPMXP1020230118); and the Nakatani Foundation.

## Supporting information


**Table S1.** IVIM model parameters (*N* = 10 000).

## Data Availability

The data that support the findings of this study are available from the corresponding author upon reasonable request.
