# Supplementary material for: A theoretical interpretation of diffusion weighted and intravoxel incoherent motion imaging for cerebrospinal fluid flow
Source: Magn Reson Med. 2025 Sep 4;95(1):628–36. doi: 10.1002/mrm.70062 (PMC12620170; doi:10.1002/mrm.70062)
Supplement: Supplementary file 1 — Table S1. IVIM model parameters (N = 10 000). [file MRM-95-628-s001.pdf]

Supplementary Material: A theoretical interpretation of diffusion weighted and intravoxel incoherent motion imaging for cerebrospinal fluid flow

T. Otani, Y. Bito, S. Yamada, Y. Watanabe, and S. Wada

SUPPLEMENTARY MATERIAL 1: IVIM MODEL FITTING

Table S1 summarizes the fit of the IVIM parameters on the signals with Rician noise (SNR = 20). The value of f_{VOF} and D_p tended to increase as α increased. There was a relatively broad range of f_{VOF} in the case of $\alpha = 0.1$, whereas the variations became smaller as α increased and f_{VOF} was almost higher than 0.9 in the case of $\alpha = 10$. However, there were large variations in D_p regardless of α, and the value of D_p was one-order higher at $\alpha = 10$. On the contrary, the value of D_d ranged from the minimum value (5×10^{-5} mm^2/s) to $\mathcal{O}(10^{-3})$ mm^2/s in all cases.

The results indicate that the IVIM parameters, especially f_{VOF} and D_p, reflect the gradients of the signal attenuations originating from the flow effects in the low-b range, while the background noise becomes dominant in high b ranges and causes considerable variations in parameter fittings, spoiling the physical significance of D_d. These findings suggest that the IVIM parameters may roughly provide the extent of intravoxel flow velocity distributions, despite the inconsistency of the original IVIM model assumptions, whereas they also indicate the difficulties of using these quantitative evaluations because of the uncertainty due to background noise.

Table S1. IVIM model parameters (N = 10000).

Parameter	α	mean± s.d.	median
f_{VOF}	0.1	0.679 ± 0.308	0.862
	1	0.888 ± 0.132	0.932
	10	0.935 ± 0.027	0.939
D_p [10^{-3} mm^2/s]	0.1	14.40 ± 71.50	4.217
	1	9.293 ± 22.92	8.141
	10	132.1 ± 762.9	47.92
D_d [10^{-3} mm^2/s]	0.1	1.416 ± 1.510	0.5705
	1	0.5157 ± 0.9577	0.05
	10	0.3377 ± 0.552	0.05
